# Measurement of surface roughness changes of unpolished and polished enamel following erosion

**DOI:** 10.1371/journal.pone.0182406

**Published:** 2017-08-03

**Authors:** Francesca Mullan, Rupert S. Austin, Charles R. Parkinson, Adam Hasan, David W. Bartlett

**Affiliations:** 1 King's College London Dental Institute, Guy’s, King’s and St.Thomas’ Hospitals, London, United Kingdom; 2 GSK Consumer Healthcare, Weybridge, United Kingdom; University of Adelaide, AUSTRALIA

## Abstract

**Objectives:**

To determine if Sa roughness data from measuring one central location of unpolished and polished enamel were representative of the overall surfaces before and after erosion.

**Methods:**

Twenty human enamel sections (4x4 mm) were embedded in bis-acryl composite and randomised to either a native or polishing enamel preparation protocol. Enamel samples were subjected to an acid challenge (15 minutes 100 mL orange juice, pH 3.2, titratable acidity 41.3mmol OH/L, 62.5 rpm agitation, repeated for three cycles). Median (IQR) surface roughness [Sa] was measured at baseline and after erosion from both a centralised cluster and four peripheral clusters. Within each cluster, five smaller areas (0.04 mm^2^) provided the Sa roughness data.

**Results:**

For both unpolished and polished enamel samples there were no significant differences between measuring one central cluster or four peripheral clusters, before and after erosion. For unpolished enamel the single central cluster had a median (IQR) Sa roughness of 1.45 (2.58) μm and the four peripheral clusters had a median (IQR) of 1.32 (4.86) μm before erosion; after erosion there were statistically significant reductions to 0.38 (0.35) μm and 0.34 (0.49) μm respectively (p<0.0001). Polished enamel had a median (IQR) Sa roughness 0.04 (0.17) μm for the single central cluster and 0.05 (0.15) μm for the four peripheral clusters which statistically significantly increased after erosion to 0.27 (0.08) μm for both (p<0.0001).

**Conclusion:**

Measuring one central cluster of unpolished and polished enamel was representative of the overall enamel surface roughness, before and after erosion.

## Introduction

Currently there are no *in vivo* diagnostic tests that can measure the activity of dental erosion, despite early detection and diagnosis being important to prevention [1–3]. A possible predictor of early erosion might be changes to enamel surface roughness, and therefore there is a need to validate if non-invasive high quality measurements have the potential to be applied *in vivo* [3].

There are inherent challenges in carrying out high-resolution characterisation of intra-oral dental hard tissue surface features. Enamel is a highly mineralised tissue, made up from 96% hydroxyapatite crystallites, 3% water and 1% organic proteins [4]. Enamel composition varies in its location, with the enamel at the gingival margin being the thinnest [5] and whilst most enamel prisms run longitudinally from the enamel-dentinal junction (DEJ) emerging perpendicular to the occlusal plane, they can also cross each other to form decussation patterns, commonly seen over cusps [6]. A study by Cuy et al [4], which combined nanoindentation and chemical characterisation of the mineral content of polished enamel using an electron microprobe found that the chemical composition [with respect to calcium and phosphorous] of enamel decreased towards the DEJ, and also identified a correlation with the surface becoming softer. Whilst in the uneroded tooth the outer surface enamel is aprismatic with intact enamel perikymata visible.

Unpolished enamel has a curved, richly textured surface, which presents challenges for digital mapping. Therefore, most erosion studies have utilised polished and flattened enamel surfaces for accurate digital mapping, however this has limited applicability for clinical studies [7–9]. A previous study suggested that the surface of unpolished enamel was more variable than polished enamel. Ganss et al [10] used a contact profilometer to measure profile tissue loss of unpolished and polished enamel following erosion. They observed that unpolished enamel was less susceptible to erosive changes than polished enamel and suggested the topographical characteristics varied over the surface. Therefore, it would be beneficial to ascertain if a sample area is representative of the whole unpolished enamel surface.

A surface is constituted of form (profile), waviness and roughness. Form is the overall shape of a surface and is commonly quantified as vertical loss or step height. Waviness is the medium wavelength band within a surface, whereas 3D areal surface roughness measurements give an indication of the nature of a surface and are deviations within the form [11]. There are different ways to quantify surface roughness, with amplitude parameters being one such method. Amplitude parameters quantify the height deviations of a measured surface, 2D parameters are calculated from a single profile [12,13] but may not be truly representative of complex surfaces such as teeth. In comparison 3D parameters are calculated from the overall surface measured and provide a robust and more balanced description of the surface. This permits stable results to be obtained. For highly accurate analysis of 3D surface roughness the scan area can be as small as 0.129 by 0.129 μm [3]. The 3D areal measurements have been reported to quantify the micro-structural surface of enamel and change during erosion *in vitro* [3,8,14–17]. There are 7 defined 3D height parameters, of which Sa was the parameter used in this study which equates to the arithmetical mean of the height deviations in the surface measured and reflects the deviation in height at each point from the arithmetic mean of the surface.

A series of *in vitro* studies have attempted to model early clinical erosion [18,19]. They initially measured 2D roughness change of polished enamel along with other techniques such as calcium analysis and reflectometry. In their earlier study, Rakhmatullina et al [18] reported that following erosion, polished enamel samples exhibited an increase in diffuse reflection and decrease in spectral reflection, concurrent with a loss in enamel surface microhardness and surface calcium, and suggested a relationship between the increase in diffuse reflection and increase in surface roughness with the progression of erosion. In a follow-up study, using native and polished enamel Rakhmatullina et al [19] reported that the increase in diffuse reflections was related to surface roughness. Other studies have used surface texture measurements to investigate the changes that occur from erosive tooth wear. Hara et al [20] used surface texture measurements to differentiate between different wear aetiologies using polished enamel, unpolished enamel and dentine samples. More recently Ranjitkar et al [21] used a combination surface texture measurement and anisotropy to identify structural differences between wear caused by erosion with wear caused by erosion and attrition. They suggested that erosive wear resulted in a more complex structure whilst wear from attrition had a less complex structure but increase in anisotropy. Atomic Force Microscopy AFM] can also be used to characterise the surface of enamel [22]. Kashkosh et al [23] used AFM to characterise surface roughness changes of bovine enamel samples after 10 minutes of erosion followed by remineralising. They identified that the surfaces became significantly rougher after erosion. However, the surfaces then became significantly smoother again after remineralization. This increased smoothness was interpreted as success of the remineralising product. These are promising steps towards developing a method to quantify pathological wear at an early stage.

The aim of this study was to determine if Sa roughness of unpolished and polished enamel varies over the surface of enamel. The null hypotheses were that surface roughness would not be uniformly distributed over the surface of unpolished and polished enamel and unaffected by erosion.

## Methods

Ten extracted human maxillary and mandibular third molars without visible signs of caries or tooth wear were collected under ethics’ approval (REC: 12/LO/1836) from London Bloomsbury Research Ethics Committee. Written consent was obtained from the participants donating teeth that were planned for extraction for clinical reasons. The extracted teeth were stored in sodium hypochlorite for a minimum of three days. The roots were removed and the crowns sectioned using a circular diamond saw (XL 12205, Benetec Ltd., London, UK) to produce 20 (4 x 4 mm) buccal enamel samples. These buccal enamel sections were randomly allocated to produce ten unpolished (curved) and ten flat (polished) enamel samples. Both groups were embedded in bisacryl composite (Protemp4 3M ESPE, Germany) using custom made mould trays. The surfaces of the unpolished samples were left untouched by the composite and were cleaned using a soft toothbrush and non-fluoridated toothpaste (Kingfisher, Norwich, UK). The smear layer was removed with ethanol. Those polished, were placed in a water-cooled rotating polishing machine following previously published protocols to produce ten optically flat samples [24].

All samples were imaged using a non-contact profilometer (NCP), Laser Confocal Displacement Meter (NCP, LT-9010M, Keyence Corporation, Japan) and motion controlled stage (Xyris 2000, Taicaan, UK), before and after erosion. Surface roughness was calculated using surface metrology software (Mountains Map, DigitalSurf, France). The NCP had a laser light source with a spot size of 2 μm and vertical resolution of 10 nm and was kept in a temperature-controlled room (22.0°C) within a specially designed unit to minimise external vibrations. Previous investigations suggest a repeatability of 1 nm measuring Sa roughness of polished enamel and 7 nm measuring unpolished enamel, based upon 30 continuous measurements. The laser was emitted onto the enamel surface and the reflected light was used to construct images of the surface. The baseline measurement error of the NCP, was measured by pre-scanning an optical flat producing a background noise of 12 nm [25].

**Fig 1:**
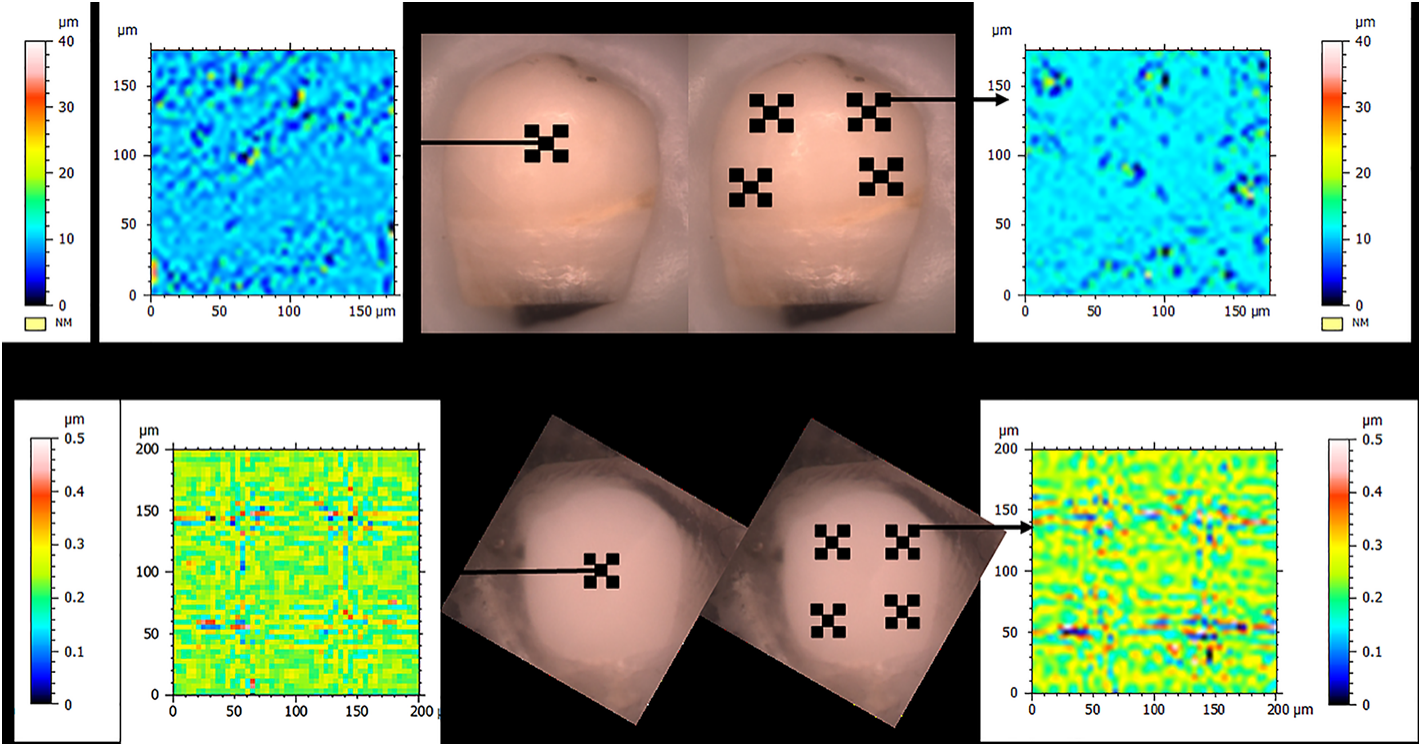
Representative diagrammatic images of an unpolished and polished enamel sample with the 5 scan areas for the central cluster and 20 scan areas for the peripheral cluster mapped out separately. A representative baseline scan image for the central cluster and peripheral cluster are also shown for each sample.

Surface roughness (Sa) was measured at baseline and after erosion in both a single central cluster (0.5 mm wide) and four equally sized peripheral clusters, each cluster approximately 1.5 mm apart and selected to represent the entire surface. Within each cluster, five smaller areas (each 200 X 200 μm) provided the Sa roughness data. The five areas within each cluster were methodically selected as shown in Fig 1. The NCP used in this study had a live video screen of the surface with a horizontal indicator line to identify the optimum focus and position the scanner. The scans from the NCP were automatically analysed using a custom designed macro program based on previously published protocols from our research group to extract Sa roughness using a Gaussian cut-off filter of 25 μm [3,26,27].

The unpolished and the polished enamel samples underwent a 3-cycle erosion at room temperature. For each cycle, unpolished or polished samples were fully immersed in 100 mL commercial orange juice (Sainsbury’s basic orange juice drink, pH 3.2, titratable acidity 41.3mmol OH/L) for 15 minutes under constant agitation at 62 rpm using an orbital shaker (Stuart Scientific, Mini Orbital Shaker S05, Bibby), with a total immersion time of 45 minutes. After one cycle, the samples were removed, rinsed with distilled water and then reinserted into the erosion cycling repeated until three cycles were completed, following which the samples were rinsed with distilled water and left to air dry for 24 hours.

One unpolished and one polished enamel sample were randomly selected for imaging with Scanning Electron Microscopy [SEM] using a Phenum ProX desktop SEM (Phenom-World BV, The Netherlands) after erosion. Five representative areas on the surface were selected similarly to the method used for the surface roughness measurements to provide a single image of the central cluster and one each of the peripheral clusters. The scan area used for the roughness measurements was 200 X 200 μm, therefore to image an area of similar size the magnification at 1100 X was selected providing a total area of 246 X 246 μm.

**Fig 2:**
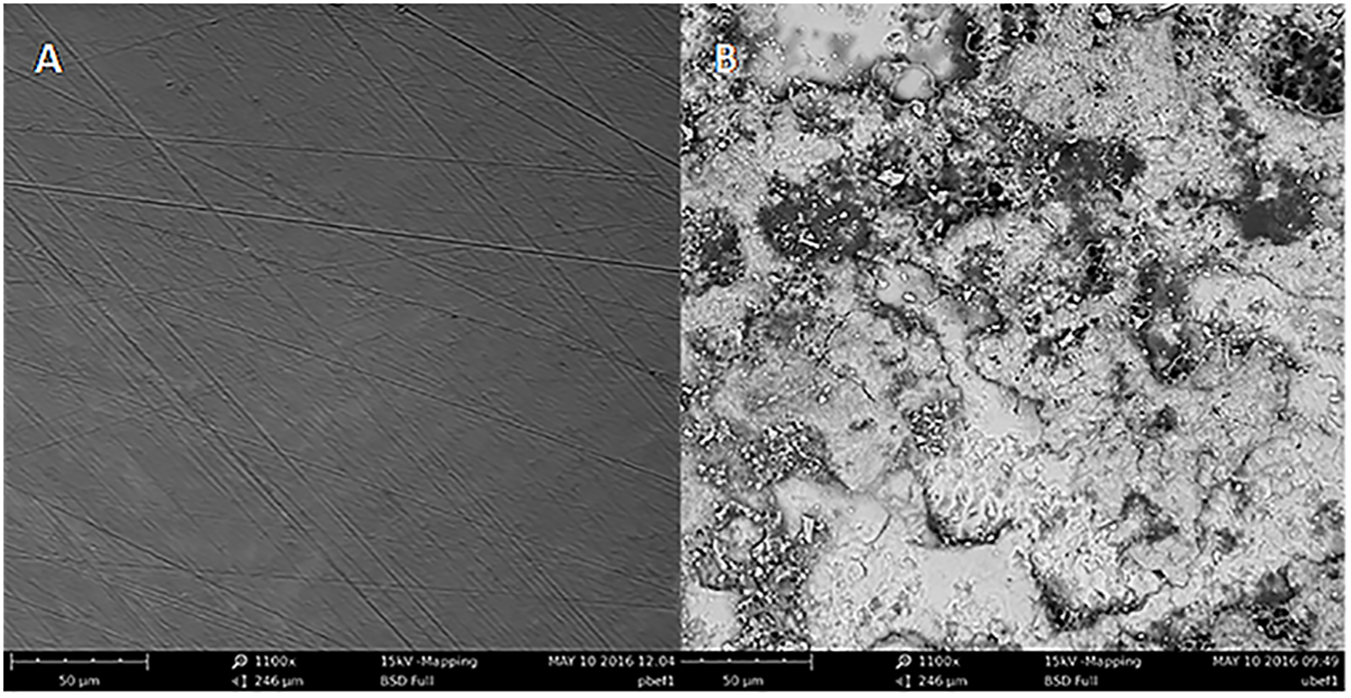
Representative SEM images before erosion. (A) Central polished enamel sample (B) Central unpolished enamel (both 1100 X magnification).

One representative peripheral SEM scan and one representative central SEM scan were selected for each. Two representative SEM images of uneroded polished and unpolished enamel samples, that were prepared in the same manner as those for this study and imaged using the same SEM and settings, were selected to aide as a comparison in Fig 2.

To determine the sample size, a calculation was carried out using G*Power3.1.92 (Heinrich-Heine-University, Dusseldorf) using mean and standard deviations based on results, from previous studies we had conducted, (mean (SD) of 0.67 (0.13) μm for unpolished enamel and 0.12 (0.02) μm for polished enamel). SPSS version 22 (IBM, United States) was used for the statistical analysis. The data were non-normally distributed. Friedman tests were used with paired Wilcoxon tests to compare the results for the singe central cluster versus the four peripheral clusters and comparing the results before erosion versus after erosion. A Bonferroni correction for multiple comparisons was carried out and significant difference was set at p<0.008. The raw and summarized data used for the statistics are in S1, S2 and S3 Files. The statistical report is in S4 File.

## Results

**Fig 3:**
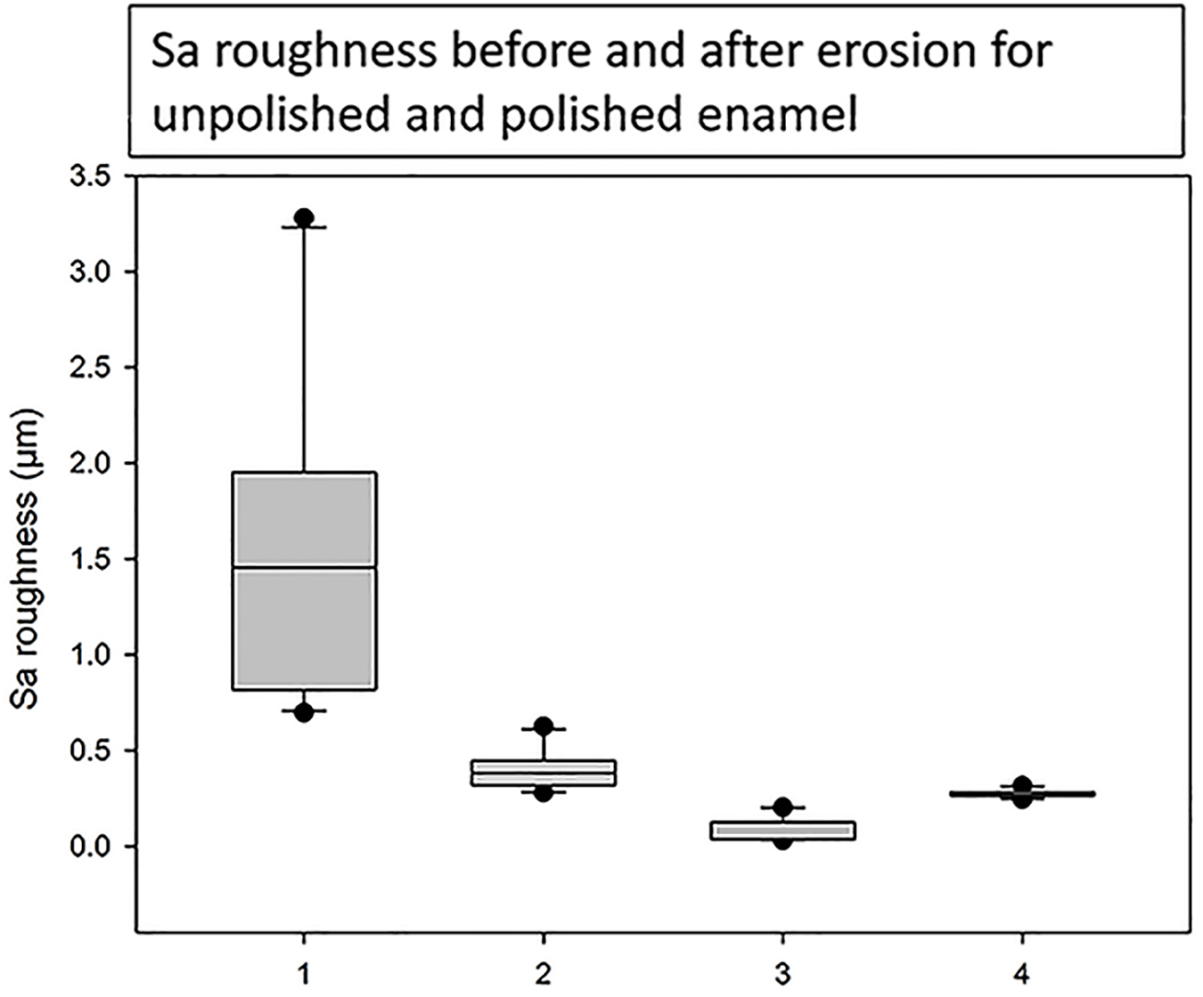
Boxplot of median (IQR) Sa roughness results of unpolished and polished enamel before and after erosion measuring 5 areas in a central cluster. 1 = unpolished enamel before erosion, 2 = unpolished enamel after erosion, 3 = polished enamel before erosion and 4 = polished enamel after erosion.

Median (IQR) was used to express the data, as the results were not normally distributed. The single central cluster for unpolished enamel had a median (IQR) Sa roughness of 1.45 (2.58) μm and the four peripheral clusters had a median (IQR) of 1.32 [4.86] μm before erosion, which reduced to 0.38 (0.35) μm and 0.34 (0.49) μm respectively after erosion (p<0.0001). For the polished enamel the Sa roughness values were 0.04 (0.17) μm measuring from the single central cluster and 0.05 (0.15) μm from the four peripheral clusters before erosion, increasing to 0.27 (0.08) μm and 0.27 (0.08) μm, respectively, after erosion (p<0.0001). Sa roughness values from the single central cluster before and after erosion for unpolished and polished enamel are expressed as a box plot in Fig 3. The unpolished samples had a relatively rougher surface before erosion with a large interquartile range, but after erosion the surface roughness and interquartile range were reduced. The polished samples had a very smooth surface and low interquartile range before erosion and whilst they became rougher after erosion, they were still smoother than the unpolished samples. The Median IQR of polished enamel after erosion was similar to that of unpolished enamel after erosion, despite unpolished enamel becoming smoother and polished enamel becoming rougher. For both unpolished and polished enamel there were no significant differences measuring the central cluster or the four peripheral clusters before or after erosion (p>0.008).

**Fig 4:**
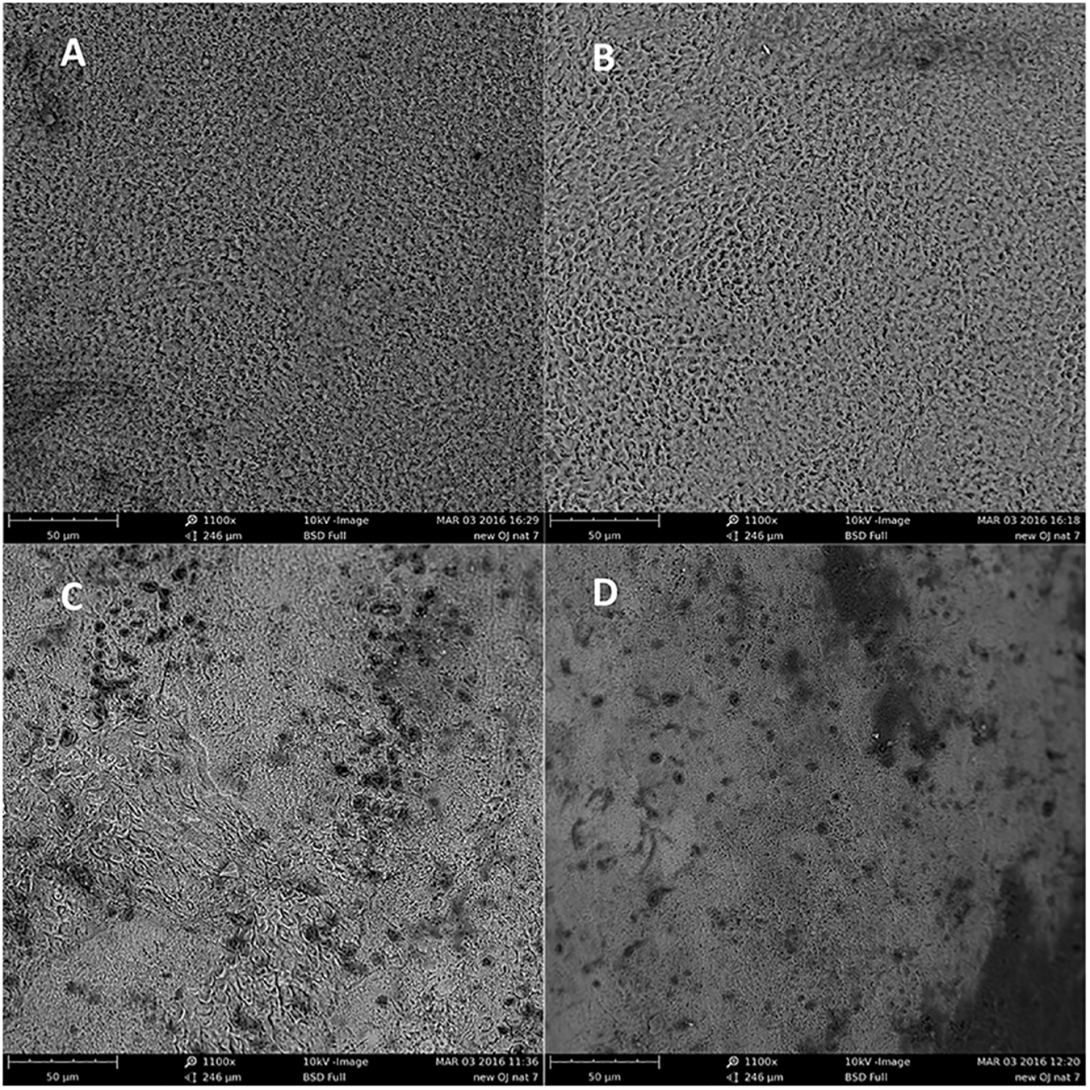
Representative SEM images after erosion. (A) Central polished enamel sample (B) Peripheral polished enamel (C) Central unpolished enamel and (D) peripheral unpolished enamel (all 1100 X magnification).

Fig 4 shows representative SEM images of the central and peripheral areas (0.06 mm^2^) of unpolished and polished enamel samples after erosion. These images revealed the presence of some similar textural features regardless of whether the images were taken from central or peripheral location for both polished and unpolished enamel following erosion. The images of polished eroded enamel clearly demonstrate the characteristic honeycomb appearance, where the core of the enamel prisms has been dissolved by acid, and the adjacent interpismatic areas appear more pronounced creating a typical appearance of type 1 enamel dissolution (Fig 4A & 4B) [28]. The images of the unpolished enamel showed a less homogenous appearance, with variations of the number of exposed prisms and varying striations of perikymata within an imaged area but with similar characteristics observed between the central cluster and peripheral clusters (Fig 4C & 4D).

## Discussion

This current study identified surface changes of unpolished enamel and polished enamel occurred after 45 minutes of erosion and that Sa roughness measurements from the centre of unpolished enamel samples and polished enamel samples were representative of the overall samples. There were no significant differences between median (IQR) Sa roughness measurements from five scan areas located in the central cluster compared to 20 scan areas in four peripheral clusters (five scan areas in each cluster) for unpolished enamel and polished enamel. The SEM images for both unpolished and polished enamel also showed similar features whether located in the central or peripheral cluster of the samples. Therefore, it is reasonable to propose that measuring a single, but, central cluster of enamel samples is sufficient to achieve representative data for the whole enamel surface and so the null hypothesis can be rejected. This study has also demonstrated that whilst polished enamel becomes significantly rougher, unpolished enamel becomes significantly smoother following exposure to dietary acid erosion.

The extracted molars used in this study may have had previous fluoride exposure as fluoride incorporation can occur either during tooth formation or after tooth eruption and the polishing protocol would have removed any fluoride benefits for these samples. However after 45 minutes of cyclical erosive challenges, any benefits the unpolished enamel samples may have had from any fluoride exposure would have been overridden [29].

The shape of a specimen influences the output when using optical tomography as flat surfaces reflect most of the light perpendicularly, whereas curved surfaces distort the light beam [30]. This curvature is visible in the SEM images of unpolished enamel where the periphery of the images is slightly out of focus. It was not possible to image the full area of an unpolished enamel sample, as the different heights over the curved surface required different focus levels; therefore, multiple smaller areas (5 X 0.04 mm^2^) were scanned. For unpolished enamel samples, the best area of focus was the apex of the curvature, which equates to the central cluster area. These relatively small surface areas for each scan area (200 X 200 μm) were chosen to minimize drop out over the curvature of the unpolished enamel samples as did using the video screen indicator to locate optimum level of focus at each region of the samples central and peripheral, based upon previous pilot work. By comparing the Sa roughness from the central cluster to peripheral areas close by, an estimate of the overall surface features of the enamel sample was possible.

Surfaces which have higher and lower step height deviations have higher Sa roughness values and therefore classed as rougher [11]. Previous erosion studies have shown that polished enamel becomes rougher after erosion which is the same pattern seen in this study however, interestingly our unpolished enamel samples became significantly smoother [3,16]. It could be suggested that this is due to polished enamel having less textural features at baseline than unpolished enamel, as was seen in the SEM images. The finding that the unpolished enamel samples became smoother after erosion may be more representative of the clinical pattern of erosion and supports previous work [3,31–33]. Comparing the representative SEM images in Figs 2 and 4 of uneroded unpolished enamel and the eroded unpolished enamel from this study there is visible evidence of a breakdown in structure. However, in this study the SEM images show the form of the surface and not the roughness. Other authors have used specialised software and stereo SEM to transform the imaged surfaces into topographical maps from which roughness parameters were calculated [34]. Our combined roughness and SEM results suggest that there has been structural breakdown at a profile level and the roughness changes we have identified are occurring within these areas of tissue loss. A recent study by Hara et al [20] investigated the use of surface texture parameters to differentiate between different wear patterns using polished and unpolished enamel and dentine samples. The authors were unable to differentiate between sound and worn lesions by measuring Sa roughness of unpolished enamel. However, there were differences compared to the methods used in our study. They immersed samples in acid four times a day for 2 minutes without agitation. As well as a reduced immersion time compared to our study, agitation can also have an effect on erosive wear. Agitation increases fluid dynamics which facilitates more tissue loss [24]. Hara et al [20] also immersed their samples in a remineralising solution which was not carried out in our study. Furthermore, the filtering used in the analysis for Sa roughness in the study by Hara et al [20] was not specified and may have influenced the outcome. Filtering is a way of extracting roughness, waviness and form error, from a measurement and the filter cut-off is the limit wavelength between waviness and roughness. Choosing the correct filter cut-off can affect the overall measurement outcome, for some materials the cut offs are defined by ISO standards but for biological materials such as human enamel a value that best separates the waviness and the roughness by using the spectral representation of the profile must be found [14,35–37]. The Gaussian filter used in this current study was 25 μm set at 5 times the feature detail of the diameter of an enamel prism.

In a study investigating surface texture parameters for polished surfaces, Austin et al [3] recommended that the lateral resolution of optical scanning equipment should be less than 2.5 μm, as above this level the prismatic features of enamel are lost. However, this present study has revealed that for unpolished enamel surfaces, the nature of the textural changes are so different that instruments with a lateral resolution below this are able to reliably characterise enamel surfaces following an erosive wear process.

The current state of the art high-resolution means measuring equipment cannot be used intra orally, however there are replica techniques that can be used for *in vivo* studies. The use of replica techniques or impressions for longitudinal studies measuring volume loss has been established in erosion studies [15,38]. Replica techniques have also been established for qualitative assessment in enamel erosion studies using SEM [39]. Goodall et al [40] validated non-contact scanning of replicas to measure surface roughness of rough and smooth surfaces using silicon impression material and acrylic replicas. The method developed in this study to measure surface roughness of unpolished enamel has the potential for future *in vivo* studies with replicas.

## Conclusion

Measuring one central cluster of unpolished and polished enamel samples to determine Sa roughness is sufficient for subsequent studies. Polished enamel becomes significantly rougher after erosion and unpolished enamel becomes significantly smoother after erosion. These observations suggest that surface roughness derived from optical profilometry at a relatively low lateral resolution, utilizing replica methodologies, may be a relevant in vivo measure of enamel erosion.

## Supporting information

S1 File Summarised and raw data for unpolished and polished enamel. (XLSX)

S2 File Expanded raw data for polished enamel. (XLSX)

S3 File Expanded raw data for unpolished enamel. (XLSX)

S4 File Statistical report. (DOCX)
