# Supplementary material for: Measurement of surface roughness changes of unpolished and polished enamel following erosion
Source: PLoS One. 2017 Aug 3;12(8):e0182406. doi: 10.1371/journal.pone.0182406 (PMC5542659; doi:10.1371/journal.pone.0182406)
Supplement: S4 File — (DOCX) [file pone.0182406.s004.docx]

All measurements are of Sa roughness and the units are µm

| SDBN1 | Summarised data before erosion of natural enamel measuring 1 cluster |
| --- | --- |
| RDBN1 | Raw data before erosion of natural enamel measuring 1 cluster |
| SDBN4 | Summarised data before erosion of natural enamel measuring 4 clusters |
| RDBN4 | Raw data before erosion of natural enamel measuring 4 clusters |
| SDAN1 | Summarised data after erosion of natural enamel measuring 1 clusters |
| RDAN1 | Raw data after erosion of natural enamel measuring 1 clusters |
| SDAN4 | Summarised data after erosion of natural enamel measuring 4 clusters |
| RDAN4 | Raw data after erosion of natural enamel measuring 4 clusters |
| SDBP1 | Summarised data before erosion of polished enamel measuring 1 cluster |
| RDBP1 | Raw data before erosion of polished enamel measuring 1 cluster |
| SDBP4 | Summarised data before erosion of polished enamel measuring 4 clusters |
| RDBP4 | Raw data before erosion of polished enamel measuring 4 clusters |
| SDAP1 | Summarised data after erosion of polished enamel measuring 1 cluster |
| RDAP1 | Raw data after erosion of polished enamel measuring 1 cluster |
| SDAP4 | Summarised data after erosion of polished enamel measuring 4 clusters |
| RDAN4 | Raw data after erosion of polished enamel measuring 4 clusters |

Natural enamel


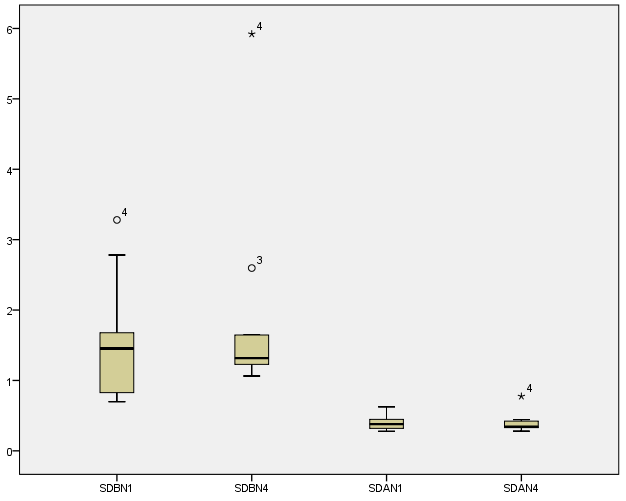


Polished enamel


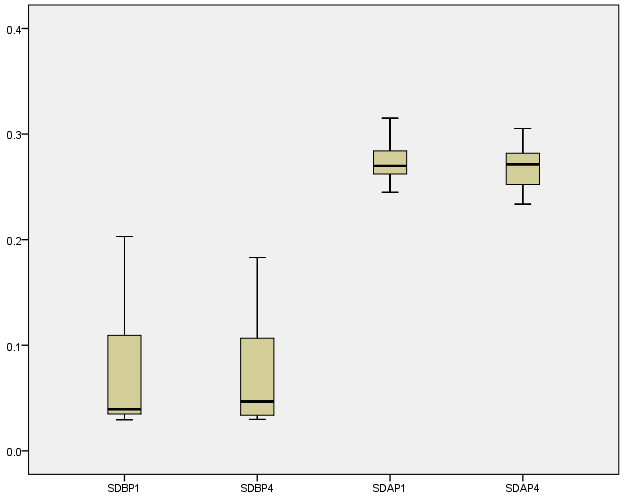


The analysis provides evidence for a difference in SA after erosion but no difference between SA from 1 cluster compared with 4 clusters. We have no evidence that this is the case in unsampled areas of the sections. The analysis for the polished enamel yields the similar conclusions.The SA values from polished enamel sections before erosion are lower compared with SA from polished enamel sections after erosion (the reverse finding for natural enamel). This is easily seen in the boxplots for natural and polished enamel sections.

The Friedman test (the equivalent of the repeated measures ANOVA has been used for these skewed data) has been used with post-hoc by multiple comparisons using paired Wilcoxon test. Bonferroni correction for multiple comparisons (for significant difference, p<0.008333)

| **Natural enamel sectionsTest Statistics^a^** | | | | | | |
| --- | --- | --- | --- | --- | --- | --- |
|  | SDBN4 - SDBN1 | SDAN4 - SDAN1 | SDAN1 - SDBN1 | SDAN4 - SDBN1 | SDAN4 - SDBN4 | SDAN1 - SDBN4 |
| Z | -1.274^b^ | -.764^c^ | -2.803^c^ | -2.803^c^ | -2.803^c^ | -2.803^c^ |
| Asymp. Sig. (2-tailed) | .203 | .445 | .005 | .005 | .005 | .005 |
| a. Wilcoxon Signed Ranks Test | | | | | | |
| b. Based on negative ranks. | | | | | | |
| c. Based on positive ranks. | | | | | | |

SDBN4 - SDBN1

| **Test Statistics^a^** | |
| --- | --- |
|  | all_Sa_data |
| Mann-Whitney U | 45.000 |
| Wilcoxon W | 100.000 |
| Z | -.378 |
| Asymp. Sig. (2-tailed) | .705 |
| Exact Sig. [2*(1-tailed Sig.)] | .739^b^ |
| a. Grouping Variable: Groupingfactor | |
| b. Not corrected for ties. | |

SDAN1 - SDBN4

| **Test Statistics^a^** | |
| --- | --- |
|  | all_Sa_data |
| Mann-Whitney U | 45.000 |
| Wilcoxon W | 100.000 |
| Z | -.378 |
| Asymp. Sig. (2-tailed) | .705 |
| Exact Sig. [2*(1-tailed Sig.)] | .739^b^ |
| a. Grouping Variable: Groupingfactor | |
| b. Not corrected for ties. | |

| **Descriptive Statistics** | | | | | | |
| --- | --- | --- | --- | --- | --- | --- |
|  | N | Mean | | Std. Deviation | Minimum | Maximum |
| SDBN1 | 10 | 1.550627931450000 | | .868026061837249 | .6965184406400000 | 3.2801710802000000 |
| SDBN4 | 10 | 1.899904460025036 | | 1.478646202817847 | 1.0634645578200002 | 5.9210976768300000 |
| SDAN1 | 10 | .393229537309000 | | .098989618894761 | .2791638042200000 | .6250248565200000 |
| SDAN4 | 10 | .397890917671385 | | .141811436612859 | .2808990048000000 | .7748093607368420 |
| **Ranks** | | |  |  |  |  |
|  | Mean Rank | |  |  |  |  |
| SDBN1 | 3.40 | |  |  |  |  |
| SDBN4 | 3.60 | |  |  |  |  |
| SDAN1 | 1.50 | |  |  |  |  |
| SDAN4 | 1.50 | |  |  |  |  |

| **Test Statistics^a^** | |
| --- | --- |
| N | 10 |
| Chi-Square | 24.120 |
| df | 3 |
| Asymp. Sig. | .000024 |
| a. Friedman Test | |

There is a statistically significant difference between the 4 groups of natural enamel sections (p<0.0001).

| **Natural enamel sections Descriptive Statistics** | | | | | | | | |
| --- | --- | --- | --- | --- | --- | --- | --- | --- |
|  | N | Mean | Std. Deviation | Minimum | Maximum | Percentiles | | |
|  |  |  |  |  |  | 25th | 50th (Median) | 75th |
| SDBN1 | 10 | 1.550627931450000 | .868026061837249 | .6965184406400000 | 3.2801710802000000 | .816236385285000 | 1.454276517300000 | 1.953663837700000 |
| SDAN1 | 10 | .393229537309000 | .098989618894761 | .2791638042200000 | .6250248565200000 | .317566124680000 | .380031097350000 | .448398850510000 |
| SDBN4 | 10 | 1.899904460025036 | 1.478646202817847 | 1.0634645578200002 | 5.9210976768300000 | 1.212627907150132 | 1.317758308270395 | 1.883738775900000 |
| SDAN4 | 10 | .397890917671385 | .141811436612859 | .2808990048000000 | .7748093607368420 | .323571292764539 | .342154036575833 | .427141357621032 |

**Polished enamel sections**

| **Descriptive Statistics** | | | | | | | | |
| --- | --- | --- | --- | --- | --- | --- | --- | --- |
|  | N | Mean | Std. Deviation | Minimum | Maximum | Percentiles | | |
|  |  |  |  |  |  | 25th | 50th (Median) | 75th |
| SDBP1 | 10 | .077596140585150 | .064075822287434 | .0294850212580000 | .2030449040000000 | .033657407017500 | .039404446881750 | .125563861437500 |
| SDBP4 | 10 | .075663020954650 | .056173505571300 | .0298345580000000 | .1830948337663158 | .033065193087500 | .046716785124342 | .119743405286875 |
| SDAP1 | 10 | .272948924385000 | .019153772896057 | .2448703421400000 | .3150899825000000 | .261517127032500 | .269838747730000 | .284567353910000 |
| SDAP4 | 10 | .268106928543997 | .021773558952940 | .2336839636800000 | .3052946514263158 | .249583793806250 | .271257945089722 | .283492624950066 |

| **Ranks** | |
| --- | --- |
|  | Mean Rank |
| SDBP1 | 1.50 |
| SDBP4 | 1.50 |
| SDAP1 | 3.50 |
| SDAP4 | 3.50 |

| **Test Statistics^a^** | |
| --- | --- |
| N | 10 |
| Chi-Square | 24.000 |
| df | 3 |
| Asymp. Sig. | .000025 |
| a. Friedman Test | |

There is a statistically significant difference between the 4 groups of polished enamel sections (p<0.0001).

| **Test Statistics^a^** | | | | | | |
| --- | --- | --- | --- | --- | --- | --- |
|  | SDBP4 - SDBP1 | SDAP1 - SDBP1 | SDAP4 - SDBP1 | SDAP1 - SDBP4 | SDAP4 - SDBP4 | SDAP4 - SDAP1 |
| Z | -.153^b^ | -2.803^c^ | -2.803^c^ | -2.803^c^ | -2.803^c^ | -.357^b^ |
| Asymp. Sig. (2-tailed) | .878 | .005 | .005 | .005 | .005 | .721 |
| a. Wilcoxon Signed Ranks Test | | | | | | |
| b. Based on positive ranks. | | | | | | |
| c. Based on negative ranks. | | | | | | |

SDBP4 - SDBP1

| **Ranks** | | | | |
| --- | --- | --- | --- | --- |
|  | Groupingfactor | N | Mean Rank | Sum of Ranks |
| all_Sa_data | 5.00 | 10 | 10.35 | 103.50 |
|  | 6.00 | 10 | 10.65 | 106.50 |
|  | Total | 20 |  |  |

| **Test Statistics^a^** | |
| --- | --- |
|  | all_Sa_data |
| Mann-Whitney U | 48.500 |
| Wilcoxon W | 103.500 |
| Z | -.113 |
| Asymp. Sig. (2-tailed) | .910 |
| Exact Sig. [2*(1-tailed Sig.)] | .912^b^ |
| a. Grouping Variable: Groupingfactor | |
| b. Not corrected for ties. | |

SDAP4 - SDAP1

| **Test Statistics^a^** | |
| --- | --- |
|  | all_Sa_data |
| Mann-Whitney U | 45.000 |
| Wilcoxon W | 100.000 |
| Z | -.378 |
| Asymp. Sig. (2-tailed) | .705 |
| Exact Sig. [2*(1-tailed Sig.)] | .739^b^ |
| a. Grouping Variable: Groupingfactor | |
| b. Not corrected for ties. | |
